# Impaired hemodynamic activity in the right dorsolateral prefrontal cortex is associated with impairment of placebo analgesia and clinical symptoms in postherpetic neuralgia

**DOI:** 10.1016/j.ibror.2020.01.003

**Published:** 2020-02-07

**Authors:** Daisuke Hibi, Kouichi Takamoto, Yudai Iwama, Shohei Ebina, Hiroshi Nishimaru, Jumpei Matsumoto, Yusaku Takamura, Mitsuaki Yamazaki, Hisao Nishijo

**Affiliations:** a Department of Anesthesiology, Faculty of Medicine, University of Toyama, Japan; b System Emotional Science, Faculty of Medicine, University of Toyama, Japan; c Department of Sport and Health Sciences, Faculty of Human Sciences, University of East Asia, Japan

**Keywords:** Postherpetic neuralgia, Chronic pain, Dorsolateral prefrontal cortex, Hemodynamic responses, Placebo analgesia

## Abstract

The dorsolateral prefrontal cortex (dlPFC) is functionally linked to the descending pain modulation system and has been implicated in top down pain inhibition, including placebo analgesia. Therefore, functions of the dlPFC may be impaired in patients with chronic pain. Postherpetic neuralgia (PHN) is one of several syndromes with chronic neuropathic pain. In the present study, we investigated possible dysfunction of the dlPFC in chronic pain using patients with PHN. In a conditioning phase, heathy controls (n = 15) and patients with PHN (n = 7) were exposed to low (LF) and high (HF) frequency tones associated with noxious stimuli: weak (WS) and strong (SS) electrical stimulation, respectively. After the conditioning, cerebral hemodynamic activity was recorded from the bilateral dlPFC while the subjects were subjected to the cue tone-noxious electrical stimulation paradigm, in which incorrectly cued noxious stimuli were sometimes delivered to induce placebo and nocebo effects. The results indicated that hemodynamic responses to the LF tone in the right dlPFC was significantly lower in patients with PHN compared to the healthy controls. Furthermore, the same hemodynamic responses in the right dlPFC were correlated with placebo effects. In addition, clinical symptoms of PHN were negatively correlated to cerebral hemodynamic responses in the right dlPFC and magnitudes of the placebo effects. The results suggest that the right dlPFC, which is closely associated with the descending pain modulation system, is disturbed in PHN.

## Highlights

• Patients with chronic pain due to postherpetic neuralgia (PHN) were used.
• Hemodynamic activity in the dorsolateral prefrontal cortex (dlPFC) was analyzed.
• PHN showed less placebo effects and smaller hemodynamic responses in the right dlPFC.
• PHN symptoms were associated with smaller placebo effects and right dlPFC responses.
• Deficits in placebo effects due to dlPFC disturbance are involved in chronic pain.

## 1 Introduction

One-third to one-half of the population suffers from chronic pain (Fayaz et al., 2016). Chronic pain seriously impairs quality of life (QOL), activities of daily living, and patients’ social and family environments (Dueñas et al., 2016). Postherpetic neuralgia (PHN) is a typical chronic neuropathic pain syndrome consisting of symptoms such as spontaneous pain, tactile allodynia, and hyperpathia, which may develop after the healing of herpes zoster eruptions. About 30% of patients with PHN are considered refractory to current treatments (Watson and Oaklander, 2002), and neural mechanisms of neuropathic pain in PHN remain under debate (Miyazaki et al., 2005; Devor, 2018). Several studies suggest that disturbance of the descending pain modulation system is involved in the development of chronic pain (see review by Ossipov et al., 2014). Moreover, a recent clinical study reported that conditioned pain modulation was disturbed in patients with PHN, and impairment in conditioned pain modulation was correlated with clinical pain symptoms (Pickering et al., 2014). These findings suggest that the pain-inhibition system is disturbed in PHN (Pickering et al., 2014).

The dorsolateral prefrontal cortex (dlPFC) has been implicated in pain inhibition through effects on the descending pain modulation system (Lorenz et al., 2003; Craggs et al., 2007; Tracey and Mantyh, 2007). Activity of the dlPFC has been shown to increase in response to cues predicting a stressful unconditioned stimulus and inhibit activity of the amygdala in response to the unconditioned stimulus (Goodman et al., 2018). Placebo analgesia is experimentally induced by presenting specific cues associated with lower pain (i.e., expectation of lower pain) (Wager and Atlas, 2015). Activity in the dlPFC in response to a cue predicting lower noxious pain was correlated with placebo effects, as well as with activity in the descending pain modulating system (Wager et al., 2004). Furthermore, gray matter density in the dlPFC was correlated to placebo analgesia (Schweinhardt et al., 2009). These findings suggest that the dlPFC plays an important role in pain inhibition.

In patients with chronic pain, it has been proposed that differential associative learning of conditioned stimuli (CSs) is impaired when CSs are differentially associated with strong/weak and noxious/non-noxious events (Schneider et al., 2004; Jenewein et al., 2013; Meulders et al., 2014, 2015; Harvie et al., 2017). These studies suggest that deficits in identifying the safe cue (i.e., CSs associated with weak noxious or non-noxious events) lead to elevated anxiety and fear. Subsequently, deficits in inhibiting fear responses in the presence of safe CSs may lead to chronic pain (Meulders et al., 2014, 2015), since pain-related fear is an important factor for development of chronic pain (Zale et al., 2013). The substantial role of the dlPFC in placebo analgesia suggests involvement of the dlPFC in associative conditioning to induce placebo effects.

Accumulating evidence suggests that the dlPFC is morphologically and functionally altered in patients with chronic pain (Seminowicz and Moayediz, 2017; Ong et al., 2019). In the present study, we hypothesized that functional alteration in the dlPFC might be associated with impairments in differential associative conditioning in patients with PHN. To investigate this idea, we analyzed hemodynamic activity in the dlPFC using near-infrared spectroscopy (NIRS) during differential associative conditioning, in which two different CSs were associated with weak and strong electrical stimulation. Herein, we report that the hemodynamic responses to the CSs associated with weak stimulation were decreased in patients with PHN, which was further associated with clinical pain symptoms and deficits in placebo effects.

## 2 Materials and methods

### 2.1 Participants

Seven patients with postherpetic neuralgia (PHN) diagnosed according to the diagnostic criteria proposed by the international association for the study of pain (IASP) were recruited from Department of Anesthesiology at the Toyama University Hospital. There were 4 male patients, 3 female patients, and the mean age of the patients was 67.29 ± 2.62 years (mean ± standard error [SE]). The inclusion criteria for the patients with PHN were: (1) patients who received appropriate antiviral treatment; (2) persistent pain lasting longer than three months after the onset of herpes zoster infection; (3) over fifty years of age. The exclusion criteria for the patients with PHN were: (1) patients had psychiatric disorders; (2) patients had neurological disorder(s) apart from PHN. Fifteen age- and sex-matched healthy controls (HC) who had no neurological or psychiatric disorders (males, 7; females, 8; mean age, 66.33 ± 1.64 years) participated in the present study. All participants were right-handed and had no cognitive impairment assessed by the Japanese version of Mini-Mental State Examination (J-MMSE) (Sugishita et al., 2016). This study followed the guidelines set forth by the Helsinki Declaration and was approved by the Ethical Committee for Human Clinical studies at University of Toyama. Written informed consent was obtained from all subjects who participated in this study.

### 2.2 Experimental procedures

First, current intensity of electrical cutaneous stimulation (painful stimuli) was determined. A conditioning session was then conducted, where subjects learned the association between two different cue tones and two different intensities of electrical stimulation in a cue tone-shock associative task. Finally, a recording session was conducted to measure the cerebral hemodynamic activity in the PFC during the cue tone-shock association task. The same pairs of cue tones and electrical stimulations as previously used in the conditioning session were presented in 75% of trials. Different combinations of the cue tones and electrical stimulations were presented (mismatch condition) in the remaining 25% of trials.

To determine current intensity of electrical stimulation, electrodes were placed on the subject’s back of the left hand, which was not affected by PHN. Electrical stimulation was delivered by 5 Hz sine-wave electrical stimulator (Neurometer, Neurotron Inc., Baltimore, Maryland, USA). While current intensity was slowly increased, the subjects were instructed to say “yes” when they felt maximum tolerable pain on a scale of 0–100 [from 0 (no pain) to 100 (maximum tolerable pain)]. Two intensities of electrical stimulation were determined based on the individual’s maximum tolerable pain threshold: 50% of the current intensity of the maximum tolerable pain (weak electrical stimulation: WS) and 90 % of the current intensity of the maximum tolerable pain (strong electrical stimulation: SS). After determination of the stimulus intensity, the subjects received each stimulus at each stimulus intensity before the experiment, and confirmed that both of the stimuli were noxious.

During the conditioning session, the subjects completed 20 trials to associate cue tones and the corresponding electrical stimulation. Each trial consisted of the presentation of a cue tone (5 s), electrical stimulation (5 s), and rest (10 s). Low-frequency (LF: 500 Hz, 60 db) and high-frequency (HF: 2000 Hz, 87db) cue tones were associated with WS and SS, respectively; 10 trials of LF cues-WS and another 10 trials of HF cue tones-SS were randomly presented. At the end of each electrical stimulation, subjects were asked to rate subjective pain intensity using a visual analog scale (VAS) [from 0 (no pain) to 100 (maximum tolerable pain)].

During the recording session, cerebral hemodynamic activity was measured by NIRS. Each trial consisted of the presentation of a cue tone (5 s), a delay period before electrical stimulation (5 s), electrical stimulation (5 s), and rest (20–30 s). To prevent prediction of cue onset by the subjects, the resting time was pseudorandomly set (mean resting time, 24.84 ± 0.71 s). A total of 32 trials were performed. These 32 trials were divided into four conditions, including mismatch conditions: LF-WS condition (13 trials), in which the LF cue tone was associated with WS (correctly cued stimulation); HF-SS condition (11 trials), in which the HF cue tone was associated with SS (correctly cued stimulation); LF-SS condition (4 trials), in which the LF cue tone was associated with SS (incorrectly cued stimulation); HF-WS condition (4 trials), in which the HF cue tone was associated with WS (incorrectly cued stimulation). Thus, the ratio of mismatched cued stimuli vs. matched cued stimuli was 1:3. The matched and mismatched cued stimuli were pseudo-randomly presented. At the end of each electrical stimulation, subjects were asked to rate subjective pain intensity using the visual analog scale.

### 2.3 Psychological data analysis

VAS pain in response to each electrical stimulation (WS or SS) were analyzed using repeated measures two-way analysis of variance (ANOVA) with the cue tone and subject group as factors. To assess placebo effects, VAS pain differences between the LF-SS and HF-SS conditions were computed for each participant, i.e., [(LF-SS) – (HF-SS)]. The placebo data in patients with PHN and HC were compared using the Mann-Whitney U test. To assess nocebo effects, VAS pain differences between the LF-WS and HF-WS conditions were computed in each subject, i.e., [(HF-WS) – (LF-WS)]. The nocebo data in patients with PHN and HC were compared using the Student's *t*-test.

### 2.4 NIRS data recording

**Fig. 1:**
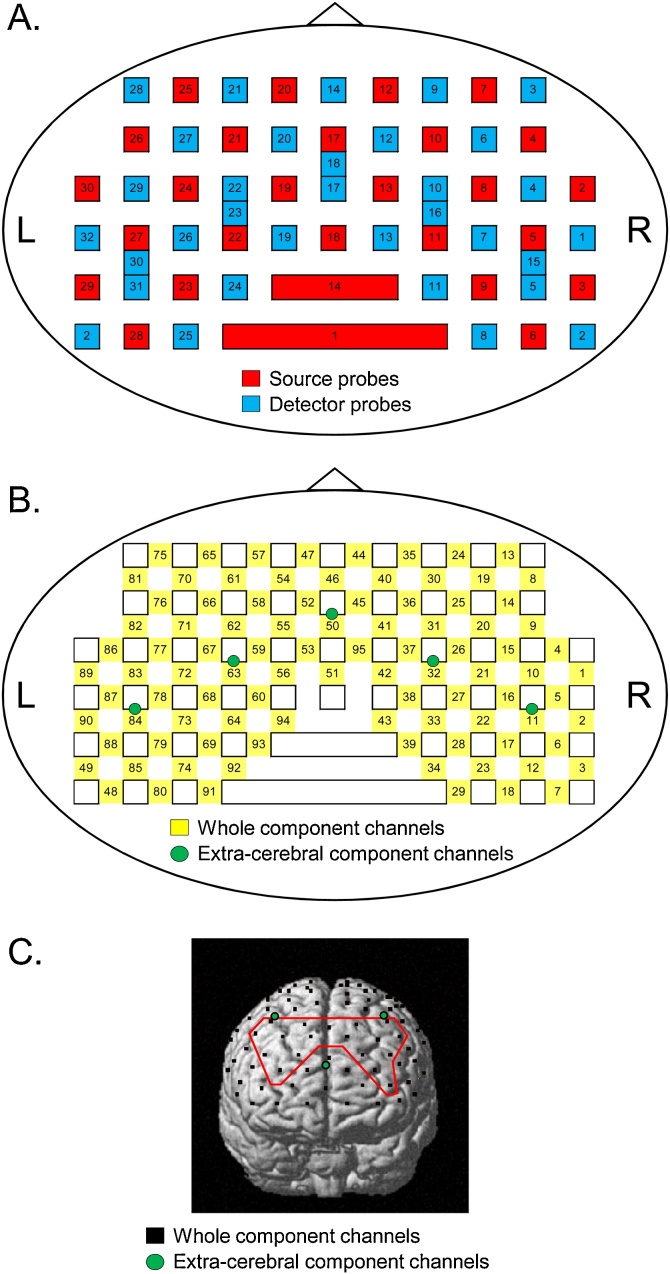
Arrangements of NIRS probes and channels. A: Arrangement of NIRS probes (source and detectors). B: Locations of NIRS channels. Green circles indicate the extracerebral hemodynamic component channels. C: Example of spatial registration of NIRS channels. Black dots indicate the whole component channels. Green dots indicate the extracerebral hemodynamic component channels. The region surrounded by the red line indicates the dorsolateral prefrontal cortex (dlPFC) (For interpretation of the references to colour in this figure legend, the reader is referred to the web version of this article.).

Cerebral hemodynamic activity was measured by two NIRS systems (OMM 3000, Shimadzu Inc., Kyoto). The NIRS head cap was placed on the heads of the subjects. The 28 source probes and 32 detector probes were placed on the head cap. The 27 detector probes were placed 3 cm away from the source probes, while 5 detector probes were placed 1.5 cm away from the source probes (Fig. 1A). The most anterior and lowest NIRS probes were placed at the FP10-FP2 line of the international 10–20 method for EEG recording (Jurcak et al., 2007). The midpoint of the source and detector probes, from which the NIRS signals were derived, were defined as NIRS channels (Fig. 1B). Hemodynamic activity was measured by light with different wave lengths (708, 805, and 830 nm). The changes in hemoglobin concentration (Oxy-Hb, Deoxy-Hb, and Total Hb) were estimated based on the modified Lambert-Beer law (Seiyama et al., 1988; Wray et al., 1988). After the experiments, the three-dimensional coordinates of the probes and NIRS channels in each subject were measured by a digitizer (FASTRAK, Polhemus Inc., USA).

### 2.5 NIRS data analysis

As NIRS signals include both cerebral and extra-cerebral hemodynamic components, we applied a multi-distance probe arrangement method to remove the extra-cerebral components from NIRS signals (Sato et al., 2016; Ishikuro et al., 2014; Saager et al., 2011; Nakamichi et al., 2018). When the distance between the source and detector probes was at 3 cm, NIRS signals include both cerebral and extracerebral (scalp, skull, and cerebrospinal fluid) hemodynamic signals (whole component signals). Additionally, NIRS signals mainly include the extracerebral hemodynamic component when the distance between source and detector probes was 1.5 cm (Fukui et al., 2003; Niederer et al., 2008). Thus, cerebral hemodynamic responses (Oxy-Hb, Deoxy-Hb, and Total-Hb) were estimated by simple-subtraction methods (Schytz et al., 2009). Cerebral components of the signals were estimated by subtracting the extra-cerebral signals located nearest to corresponding whole component signals from the whole component signals, i.e., [(whole component signals) – (extra-cerebral signals)]. To remove physiological noises due to respiration, cardiac activity, and baseline drifts, the band pass filter was set at 0.01 Hz to 0.1 Hz (Tong et al., 2011; Yasumura et al., 2014). NIRS signals in each trial were summed and averaged to analyze hemodynamic responses to the cue tones. Averaged responses were corrected for the mean baseline activity from -8 to - 5 s before the onset of the cue tones.

Since Oxy-Hb best reflects brain activity (Hoshi et al., 2001), Oxy-Hb data were analyzed to compare hemodynamic responses during presentation of auditory cues between patients with PHN and HC. Effect sizes of hemodynamic responses to the cue tones were calculated according to the following formula: effect size = [(mean Oxy-Hb levels for 10 s from cue tone onset) – (mean Oxy-Hb levels during the baseline period from -8 to -5 s before cue tone onset)] / [standard deviation of Oxy-Hb levels during the baseline period from -8 to -5 s before cue tone onset].

To identify the anatomical location in each channel, three-dimensional coordinates of the NIRS channels in each subject were normalized to the MNI coordinates using virtual registration (Tsuzuki et al., 2007). Subsequently, locations of NIRS channels in each Brodmann area (BA) were estimated using the MRIcro software (http://www.MRIcro.com) in each subject (Fig. 1C). In the present study, we defined the regions of interest (ROIs) as the right and left dorsolateral prefrontal cortex (dlPFC: BA9, 46). The effect sizes of the Oxy-Hb data derived from the channels within each ROI were averaged in each ROI in each subject.

Hemodynamic responses (effect sizes) in the dlPFC to LF cue tones were analyzed using repeated measures two-way analysis of variance (ANOVA) with ROI and subject group as factors. When HF cue tones were presented, the data did not show normal distribution (see below), and hemodynamic responses to HF cue tone were compared between HC and patients with PHN using the Kruskal-Wallis test.

### 2.6 Correlation analysis

Correlations among NIRS responses in the dlPFC, clinical symptoms (numerical rating scale, NRS), and psychological data (placebo and nocebo effects) were analyzed using simple regression analysis. In these analyses, NRS = 0 was used for the clinical symptoms in the HC subjects.

### 2.7 Statistical analysis

All of the data are presented as mean ± standard error. All data were tested for normal distribution using the Kolmogorov-Smirnov test. Homogeneity of variance for repeated measures two-way ANOVA was evaluated using Mauchly’s test of sphericity. The baseline characteristics of the subjects (age, current intensity of electrical stimulation, and MMSE) were compared between patients with PHN and HC using the independent Student’s *t*-test or the Mann-Whitney U test. The chi-square test was used to compare gender between patients with PHN and HC. All statistical analysis was performed using SPSS (IBM Inc., New York, USA). The statistical significance level was set at P < 0.05.

## 3 Results

### 3.1 Baseline characteristics

Table 1 shows clinical characteristics of the patients with PHN, who had histories of chronic pain ranging from 3 to 45 months. Table 2 shows the baseline characteristics in patients with PHN and HC. There were no significant differences in age, sex, current intensity for maximum tolerable pain, or MMSE scores of the base line characteristics between patients with PHN and HC (Student’s *t*-test and χ^2^ test, P > 0.05).

**Table 1:** Clinical characteristics of patients with PHN.

Patient ID	Age (years)	Gender (M/F)	Affected nerve(s)	Pain duration (months)	NRS scores	Medication
1	67	M	Left V1	11	8	pregabalin, duloxetine, toramadol acetaminophen
2	58	M	Left T5	12	1	pregabalin, duloxetine, keishikajutsubuto
3	78	F	Left T6-9	45	2	mexiletine, pregabalin, duloxetine, toramadol acetaminophen, hochuekkito
4	57	F	Right T8-9	3	3	pregabalin, nortriptyline
5	71	F	Right T7-8	41	2	duloxetine, toramadol acetaminophen, keishikajutsubuto
6	69	M	Left T11-12	12	1	toramadol acetaminophen
7	71	M	Right T6-7	22	7	pregabalin, duloxetine, toramadol acetaminophen

PHN, postherpetic neuralgia; M, male; F, female; V1, V1 division of the trigeminal nerve; T, level of thoracic vertebrae; NRS, numerical rating scale.

**Table 2:** Demographic and clinical features of the subjects.

	Patients with PHN (n = 7)	HC (n = 15)	p-value
Gender (male/female)	4/3	7/8	0.647 (chi-square test)
Age (years)	67.29 ± 2.62	66.33 ± 1.64	0.779 (Student's t-test)
Current intensity of stimulation (mA)	2.11 ± 0.51	2.14 ± 0.34	0.949 (Student's t-test)
MMSE	29 ± 0.64	28.9 ± 0.31	0.477 (Mann-Whitney U test)

Values are means ± standard error (SE). PHN, postherpetic neuralgia; HC, healthy controls; MMSE, mini-mental state examination.

### 3.2 Changes in subjective pain perception

**Fig. 2:**
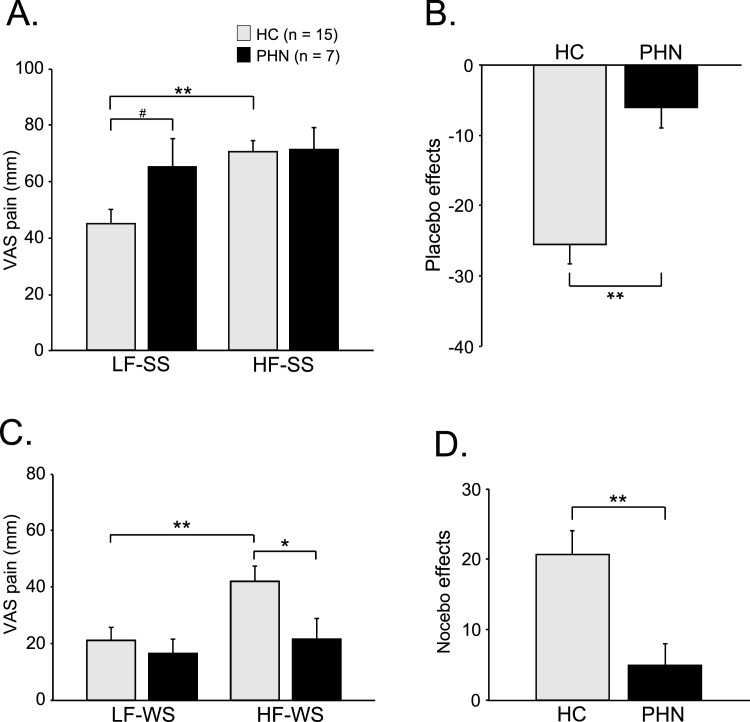
Subjective pain perception in HC and PHN. A: Comparison of VAS pain when SS was delivered. B: Comparison of VAS pain difference between the LF-SS and HF-SS conditions (i.e., placebo effects) between HC and PHN. C: Comparison of VAS pain when WS was delivered. D: Comparison of VAS pain difference between the HF-WS and LF-WS conditions (i.e., nocebo effects) between HC than patients with PHN. Error bars indicate the SEM. VAS, visual analog scale; HC, healthy controls; PHN, postherpetic neuralgia; LF-SS, strong electrical stimulation (SS) after low frequency (LF) cue tone; HF-SS, strong electrical stimulation after high frequency (HF) cue tone; LF-WS, weak electrical stimulation (WS) after low frequency (LF) cue tone; HF-WS, weak electrical stimulation (WS) after high frequency (HF) cue tone. *, **, P < 0.05, 0.01, respectively. # indicates marginal significance (P = 0.054).

Fig. 2 shows the comparisons of subjective pain scores (VAS pain) in each condition between patients with PHN and HC. When SSs were delivered (Fig. 2A), repeated measures two-way ANOVA (condition x subject group) revealed a significant main effect of condition [F (1, 20) = 48.53, P < 0.01] and a significant interaction between condition and subject group [F (1, 20) = 18.41, P < 0.01]. However, there was no significant main effect of subject group [F (1,20) = 1.46, P > 0.05] (Fig. 2A). Post-hoc comparisons indicated that VAS pain was significantly lower in the LF-SS condition compared with the HF-SS condition in HC (P < 0.01, Bonferroni test). In the LF-SS (mismatched) condition, VAS pain tended to be lower in HC than in patients with PHN (P = 0.054, Bonferroni test). The VAS pain differences between the LF-SS and HF-SS conditions (i.e., placebo effects) were significantly greater in HC than in patients with PHN, which shows a greater pain reduction in HC (Student’s *t*-test, P < 0.01) (Fig. 2B). When WSs were delivered (Fig. 2C), repeated measures two-way ANOVA (condition x subject group) revealed a significant main effect of condition [F (1,20) = 22.23, P < 0.01] and a significant interaction between condition and subject group [F (1, 20) = 8.28, P < 0.01]. However, there was no significant main effect of subject group [F (1,20) = 2.54, P > 0.05]. Post-hoc comparisons indicated that VAS pain was significantly higher in the HF-WS condition than the LF-WS condition in HC (P < 0.01, Bonferroni test). In the HF-WS (mismatched) condition, VAS pain was significantly higher in HC than in patients with PHN (P < 0.05, Bonferroni test). The VAS pain differences between HF-WS and LF-WS conditions (i.e., nocebo effects) were significantly greater in HC than in patients with PHN (Student’s *t*-test, P < 0.01) (Fig. 2D).

### 3.3 Hemodynamic responses in the PFC

**Fig. 3:**
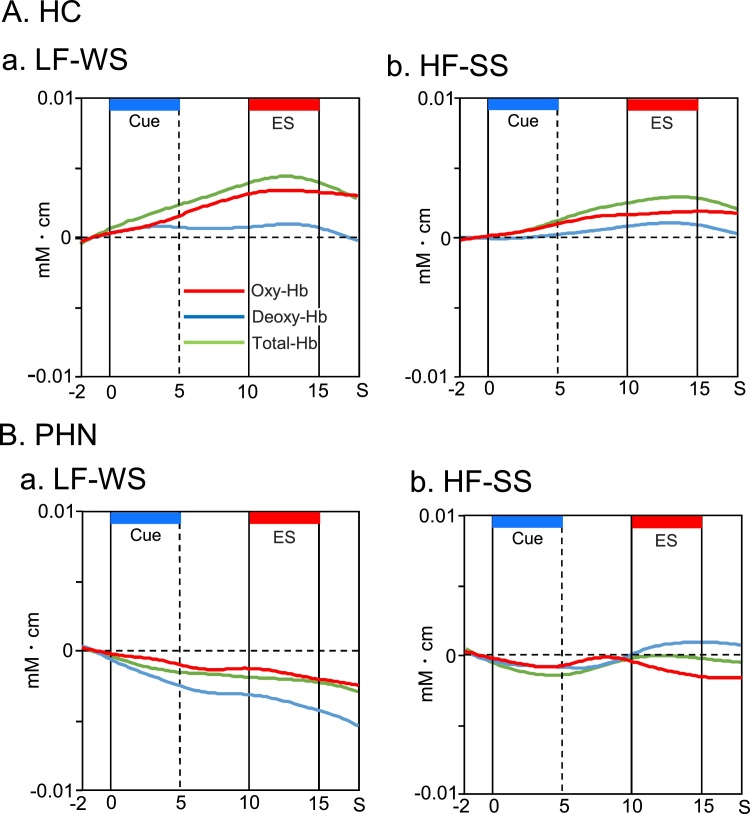
Examples of temporal changes of cerebral hemodynamic responses in the r-dlPFC during the associative conditioning task in HC (A) and patients with PHN (B). Hemodynamic responses were recorded from Ch 36 [MNI coordinates: (42, 56, 20) mm] in a healthy control (HC) and Ch 36 [MNI coordinates: (41,40,40)] in a patient with postherpetic neuralgia (PHN). Red, blue, green lines indicate Oxy-Hb, Deoxy-Hb, and Total-Hb, respectively. ES, electrical stimulation. LF-WS, low frequency (LF) cue tone followed by weak electrical stimulation (WS); HF-SS, high frequency (HF) cue tone followed by strong electrical stimulation (SS) (For interpretation of the references to colour in this figure legend, the reader is referred to the web version of this article.).

**Fig. 4:**
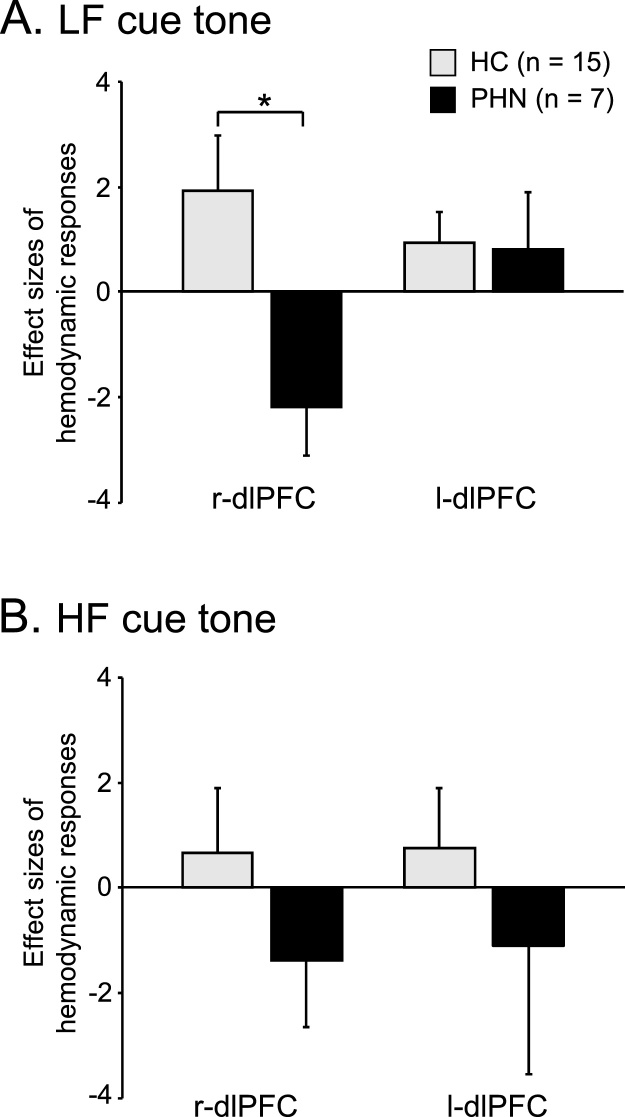
Comparison of effect sizes of hemodynamic responses in the r- and l-dlPFC to the LF (A) and HF (B) cue tones. *, P < 0.05.

Fig. 3 shows typical temporal changes of cerebral hemodynamic responses in the r-dlPFC in the cue tone-shock associative task in HC (Fig. 3A) and in patients with PHN (Fig. 3B). In HC, Oxy-Hb concentration was gradually increased during and after presentation of the cue tones in both LF-WS and HF-SS conditions (Fig. 3A). In patients with PHN, Oxy-Hb concentration was gradually decreased during and after cue tone presentation in both LF-WS and HF-SS conditions (Fig. 3B). Fig. 4 shows comparisons of mean effect sizes of hemodynamic responses (Oxy-Hb) in the dlPFC during each condition of the task. When the LF cue tone was presented (Fig. 4A), repeated measures two-way ANOVA (ROI x subject group) revealed a significant interaction between ROI and subject group [F (1, 20) = 6.07, P < 0.05]. There were no significant main effects of ROI [F (1, 20) = 1.53, P > 0.05] or subject group [F (1, 20) = 3.27, P > 0.05]. Post-hoc comparison indicated that the mean effect sizes in the r-dlPFC were significantly greater in HC than in patients with PHN (P < 0.05, Bonferroni test). When the HF cue tone was presented (Fig. 4B), there were no significant differences in the mean effect sizes of hemodynamic responses between HC and patients with PHN in the bilateral dlPFC (P > 0.05, Kruskal-Wallis test).

We also analyzed mean effect sizes of Deoxy-Hb responses in the dlPFC during each condition of the task. However, no significant differences were observed between the two groups. Data from one sample in HC were removed as outliers based on Kolmogorov-Smirnov test, and the remaining data of Deoxy-Hb were analyzed by repeated measures two-way ANOVA (ROI x subject group). When the LF cue tone was presented, the results indicated no significant interaction between ROI and subject group [F (1, 19) = 0.55, P > 0.05]. There were no significant main effects of ROI [F (1, 19) = 1.48, P > 0.05] and subject group [F (1, 19) = 4.22, P > 0.05]. When the HF cue tone was presented, the results revealed no significant interaction between ROI and subject group [F (1, 19) = 2.37, P > 0.05]. There were no significant main effects of ROI [F (1, 19) = 1.57, P > 0.05] or subject group [F (1, 19) = 0.47, P > 0.05].

### 3.4 Relationships among subjective pain, hemodynamic responses, and clinical symptoms

**Fig. 5:**
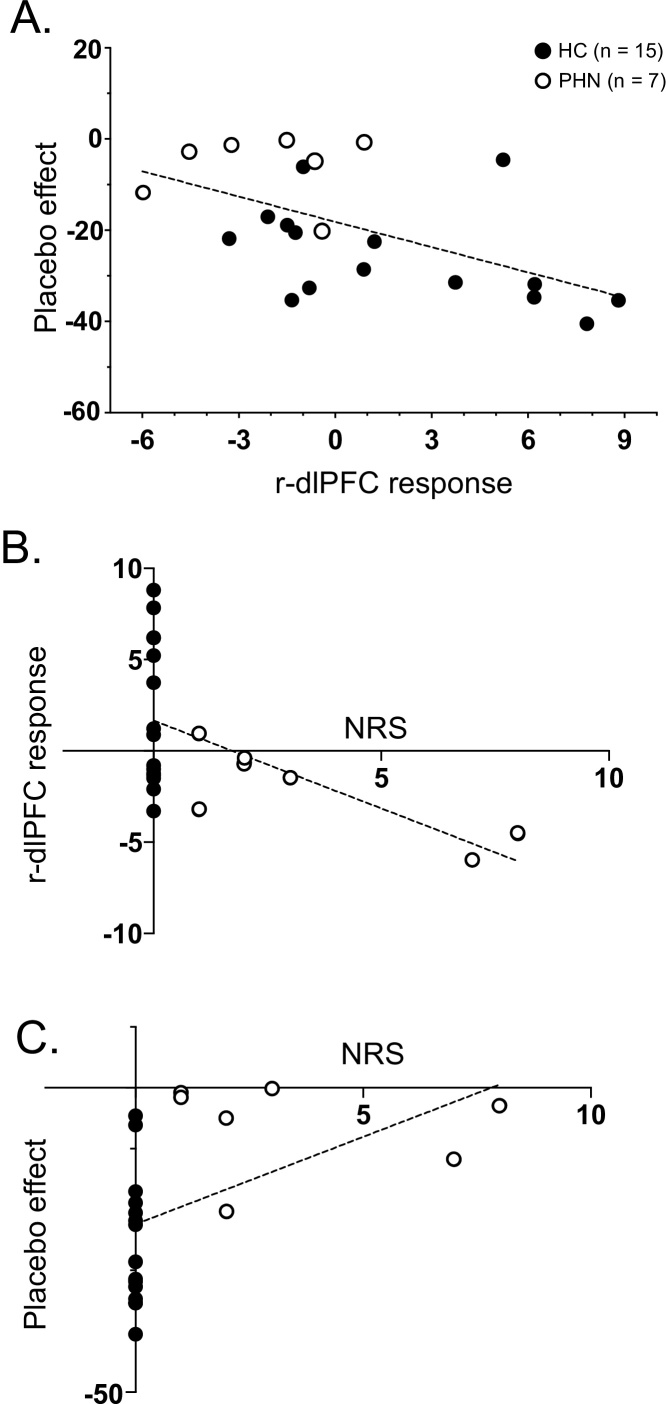
Relationships among subjective pain, hemodynamic responses in the r-dlPFC, and clinical symptoms. A: Negative correlations between VAS pain difference between the LF-SS and HF-SS conditions (placebo effects: VAS pain in the LF-SS condition minus that in the HF-SS condition) and effect sizes of hemodynamic responses to the LF cue tone in the r-dlPFC (r-dlPFC response). B: Inverse relationships between the clinical symptoms (NRS) and placebo effects. C: Positive correlation between NRS and placebo effects. VAS, visual analog scale; LF-SS, strong electrical stimulation (SS) after low frequency (LF) cue tone; HF-SS, strong electrical stimulation after high frequency (HF) cue tone.

Fig. 5A shows the relationship between VAS pain differences of the LF-SS and HF-SS conditions (placebo effects) and hemodynamic responses to LF cue tone (effect sizes of Oxy-Hb concentration) in the r-dlPFC. A statistical analysis by simple linear regression indicated that placebo effects were significantly and negatively correlated with hemodynamic responses in the r-dlPFC [r^2^ = 0.313, F (1, 20) = 9.12, P < 0.01], where larger pain reduction by placebo effects was associated with larger hemodynamic responses in the r-dlPFC. However, there was no significant correlation between placebo effects and hemodynamic responses to LF cue tone in the l-dlPFC [F (1, 20) = 0.16, P > 0.05]. Furthermore, there were no significant correlations between nocebo effects (VAS pain difference between the LF-WS and HF-WS conditions) and hemodynamic responses to HF cue tone in the r-dlPFC [F (1, 20) = 0.004, P > 0.05], nor in the l-dlPFC [F (1, 20) = 0.26, P > 0.05].

Fig. 5B shows the relationship between clinical symptoms (NRS) and hemodynamic responses to the LF cue tone in the r-dlPFC. A statistical analysis by simple linear regression indicated that clinical symptoms were significantly and negatively correlated with hemodynamic responses in the r-dlPFC [r^2^ = 0.285, F (1, 20) = 7.96, P < 0.05]. Spearman nonparametric correlation test also revealed that there was a significant and negative correlation between the clinical symptoms and hemodynamic responses in the r-dlPFC (r_s_ = -0.476, P < 0.05). Fig. 5C shows the relationship between clinical symptoms (NRS) and placebo effects. A statistical analysis by simple linear regression indicated that clinical symptoms were significantly and positively correlated with placebo effects [r^2^ = 0.233, F (1, 20) = 6.07, P < 0.05], where larger pain reduction by placebo effects was associated with less clinical symptoms. Spearman nonparametric correlation test also revealed that there was a significant and positive correlation between the clinical symptoms and placebo effects (r_s_ = -0.476, P < 0.05).

## 4 Discussion

Previous studies reported a dlPFC involvement in placebo effects (pain inhibition) in response to CSs associated with safe stimuli (weak or non-noxious stimuli) through its effects on the descending pain modulation system in heathy subjects (see Introduction). It has been proposed that recognition of CSs associated with safe stimuli is impaired in patients with chronic pain, suggesting that such deficits in safe-cue recognition lead to development of chronic pain (see Introduction). These findings suggest that the dlPFC might be disturbed in patients with chronic pain. However, responsiveness of the dlPFC to CSs is unknown in patients with chronic pain. In the present study, we analyzed dlPFC hemodynamic activity during presentation of CSs associated with weak and strong noxious stimuli in the PHN patients with chronic pain, and reported that hemodynamic (Oxy-Hb) responses in the r-dlPFC, which were correlated with placebo effects, were decreased and inversely associated with clinical symptoms in the PHN patients. The present results provide the first report of hemodynamic responses in the dlPFC during presentation of CSs to induce placebo effects in PHN patients with chronic pain.

### 4.1 Associative conditioning in PHN

In the present study, patients with PHN showed smaller placebo and nocebo effects. Since placebo and nocebo effects are attributed to associative conditioning to CSs associated with unconditioned stimuli (i.e., expectation of unconditioned stimuli;Wager and Atlas, 2015), these results suggest that differential learning between the CSs associated with WS and SS was impaired in patients with PHN. Furthermore, there was no significant difference in MMSE scores between patients with PHN and HC. This indicates that general cognitive functions in the patients with PHN were not different those in the HC, suggesting that this impairment in associative conditioning was not ascribed to general cognitive impairments due to aging in patients with PHN. Previous studies examining patients with other types of chronic pain also reported similar impairments in associative conditioning in patients with post-traumatic stress disorder with chronic pain (Jenewein et al., 2016), fibromyalgia (Becker et al., 2011; Jenewein et al., 2013; Meulder et al., 2015), and chronic hand pain (Meulder et al., 2014). These findings suggest that patients with PHN may have impairments in higher brain functions, which is similar to patients with other types of chronic pain.

We also showed that placebo effects were negatively correlated with clinical symptoms (NRS), where a larger reduction of VAS pain (placebo effects) was associated with a smaller clinical pain. Consistently, placebo effects were lower in patients with long-term fibromyalgia pain (Kosek et al., 2017) and expectation of analgesia was positively correlated with medication outcome in patients with chronic pain (Cormier et al., 2016). These findings support the hypothesis that impairments in differential associative conditioning (i.e., reduction of placebo effects) lead to chronic pain.

### 4.2 Neural mechanisms of chronic pain in PHN

In the present study, placebo effects were negatively correlated with hemodynamic responses to the CS associated with WS in the r-dlPFC, where a larger VAS pain reduction was associated with larger hemodynamic responses in the r-dlPFC. These findings are consistent with a previous study using heathy controls, in which responses in the bilateral dlPFC were correlated with placebo effects (Wager et al., 2004). Furthermore, previous studies reported that anodal (i.e., excitatory) stimulation of the r-dlPFC by direct current stimulation (tDCS) facilitated placebo effects (Egorova et al., 2015), while low frequency (i.e., inhibitory) stimulation of the r-dlPFC by repetitive transcranial magnetic stimulation (rTMS) suppressed placebo effects (Krummenacher et al., 2010).

The present study further indicated that hemodynamic responses to the CS associated with WS in the r-dlPFC were opposite between patients with PHN and HC, which was consistent with smaller placebo effects in patients with PHN than HC. Furthermore, hemodynamic responses to the CS associated with WS in the r-dlPFC were negatively correlated with clinical pain symptoms (NRS). These findings suggest that smaller placebo effects and greater clinical pain symptoms in patients with PHN may be ascribed to functional impairments in the r-dlPFC (i.e., decreased responsiveness to the safe cue). It has been reported that there were individual differences in placebo analgesia, which was associated with individual differences in anticipatory activity in the brain, including the dlPFC (Wager et al., 2011). Furthermore, lower activity of the pain-inhibition system is a risk factor for development of chronic pain (Yarnitsky et al., 2008; Lötsch et al., 2017). These findings suggest that lower responsiveness to anticipatory cues in the r-dlPFC may be one of the predisposing factors for PHN onset. These findings further suggest that the dlPFC could be a possible target for treatment of chronic pain, including PHN (Seminowicz and Moayedi, 2017). Consistent with this idea, excitatory stimulation of the r-dlPFC by tDCS and rTMS has suppressive effects on pain perception (Nahmias et al., 2009; Mylius et al., 2012). Furthermore, a recent study also reported that excitatory stimulation of the dlPFC by tDCS was effective to suppress pain in elderly subjects of the same age as in the present study (Deldar et al., 2019).

Although larger nocebo effects were observed in HC than in patients with PHN, there were no significant correlations between nocebo effects and hemodynamic responses to the CS associated with SS in the r- and l-dlPFC. Additionally, there were no correlations between nocebo effects and hemodynamic responses to the CS associated with WS in the r- and l-dlPFC in the present study. These findings suggest that anticipatory activity in the dlPFC is more closely associated with placebo effects rather than nocebo effects. Indeed, previous studies reported that nocebo effects were associated with other brain regions, such as the medial system of the pain matrix, which includes the insula cortex, operculum, anterior cingulate cortex, etc. (Kong et al., 2008; Rodriguez-Raecke et al., 2010).

### 4.3 Limitations

The present study has several limitations. In the present study, all of the patients with PHN had been prescribed medicines, such as tramadol acetaminophen, pregabalin, duloxetine, etc., after infection of herpes zoster. Human neuroimaging studies have reported that the administration of these analgesic medicines affected activity in the pain-related brain regions (primary and secondary somatosensory cortices, supplementary motor cortex, insula, anterior cingulate cortex, etc.), but without affecting the dlPFC in chronic pain patients (van Marle et al., 2011; Aupperle et al., 2012; Yue and Collaku, 2018; Wanigasekera et al., 2018). One patient had been prescribed nortriptyline, which has been shown to inhibit responses to a painful stimulus in the anterior cingulate cortex (Forcelini et al., 2014). These results suggest that the disturbed cerebral hemodynamic responses in the dlPFC in patients with PHN were not directly ascribed to administration of these analgesic medicines.

Second, decreased responses in the dlPFC in patients with PHN may be ascribed to PFC atrophy, which could be a secondary consequence of chronic pain (Ong et al., 2019). However, this atrophy due to chronic pain may be ascribed to a decrease in water content in the gray matter, but not to structural damages. The water content in the gray matter could be recovered by treatment of chronic pain (Rodriguez-Raecke et al., 2013; Pomares et al., 2017). Furthermore, there were no differences in MMSE scores between patients with PHN and HC in the present study. These findings suggest that effects of secondary cortical atrophy due to chronic pain may be not substantial in the present samples of patients with PHN. Additionally, the number of patients with PHN was relatively small in the present study.

Third, we evaluated Oxy-Hb as cerebral hemodynamic responses in the present study, since 1) signal-to-noise ratio of Deoxy-Hb is lower than Oxy-Hb (Sato et al., 2016), and 2) Deoxy-Hb is sensitive not only to venous blood oxygenation but also to venous blood volume (Hoshi et al., 2001). Consequently, the direction of changes in Deoxy-Hb were variable across tasks and individuals while direction of changes in Oxy-Hb was consistent (Hoshi et al., 2001; Toichi et al., 2004; Sato et al., 2005). Consistently, no significant results were observed in the present analysis of the Deoxy-Hb data. However, Oxy-Hb is more susceptible to systemic changes in blood circulation than Deoxy-Hb, and could yield false positive data (Tachtsidis and Scholkmann, 2016). Therefore, the extra-cerebral (systemic) signals were subtracted from the whole component signals in the present study. However, it is possible that the present subtraction method might not correct whole effects of the extra-cerebral components. Future studies with new methods, including broadband NIRS to monitor oxidation state of cytochrome-c-oxidase in the cortex (Bale et al., 2016; de Roever et al., 2017) and simultaneous monitoring of NIRS and systemic physiological variables (Scholkmann et al., 2017), could reduce false positive findings.

Finally, due to technical limitations of NIRS, we did not investigate other deep brain regions involved in placebo analgesia, such as the rostral anterior cingulate cortex, orbitofrontal cortex, nucleus accumbens, periaqueductal gray, etc. (Petrovic et al., 2002; Wager and Atlas, 2015). Further studies on the entire brain with a larger number of patients with PHN who were prescribed less medication and patients with other types of chronic pain are required to investigate a PFC role in chronic pain.

## Ethics statement

This study followed the guidelines set forth by the Helsinki Declaration and was approved by the Ethical Committee for Human Clinical studies at University of Toyama. Written informed consent was obtained from all subjects who participated in this study.

## Conflicts of interest

All authors have no conflict of interest to report.

## CRediT authorship contribution statement

**Daisuke Hibi:** Investigation, Visualization, Writing - original draft. **Kouichi Takamoto:** Investigation, Visualization, Writing - original draft. **Yudai Iwama:** Investigation, Visualization. **Shohei Ebina:** Investigation, Visualization. **Hiroshi Nishimaru:** Writing - review & editing. **Jumpei Matsumoto:** Writing - review & editing. **Yusaku Takamura:** Writing - review & editing. **Mitsuaki Yamazaki:** Conceptualization, Methodology, Supervision, Project administration, Writing - review & editing. **Hisao Nishijo:** Conceptualization, Methodology, Supervision, Project administration, Writing - review & editing.
